# Insights from Second-Line Treatments for Idiopathic Dilated Cardiomyopathy

**DOI:** 10.3390/jcdd4030012

**Published:** 2017-08-23

**Authors:** Marco Luciani, Federica del Monte

**Affiliations:** 1 Department of Cardiovascular Sciences, Università Cattolica del Sacro Cuore, Largo A. Gemelli, 8, 00168 Rome, Italy; 2 Department of Medicine, Medical University of South Carolina, Charleston, SC 29425, USA; delmonte@musc.edu

**Keywords:** idiopathic dilated cardiomyopathy, clinical trials, drug repurposing

## Abstract

Background: Dilated cardiomyopathy (DCM) is an independent nosographic entity characterized by left ventricular dilatation and contractile dysfunction leading to heart failure (HF). The idiopathic form of DCM (iDCM) occurs in the absence of coronaropathy or other known causes of DCM. Despite being different from other forms of HF for demographic, clinical, and prognostic features, its current pharmacological treatment does not significantly diverge. Methods: In this study we performed a Pubmed library search for placebo-controlled clinical investigations and a post-hoc analysis recruiting iDCM from 1985 to 2016. We searched for second-line pharmacologic treatments to reconsider drugs for iDCM management and pinpoint pathological mechanisms. Results: We found 33 clinical studies recruiting a total of 3392 patients of various durations and sizes, as well as studies that tested different drug classes (statins, pentoxifylline, inotropes). A metanalysis was unfeasible, although a statistical significance for changes upon treatment for molecular results, morphofunctional parameters, and clinical endpoints was reported. Statins appeared to be beneficial in light of their pleiotropic effects; inotropes might be tolerated more for longer times in iDCM compared to ischemic patients. General anti-inflammatory therapies do not significantly improve outcomes. Metabolic and growth modulation remain appealing fields to be investigated. Conclusions: The evaluation of drug effectiveness based on direct clinical benefit is an inductive method providing evidence-based insights. This backward approach sheds light on putative and underestimated pathologic mechanisms and thus therapeutic targets for iDCM management.

## 1. Introduction

Cardiomyopathies are a heterogenous group of diseases directly affecting the myocardial tissue composing a remarkable percentage of heart failure (HF) cases. Dilated cardiomyopathy (DCM) is defined by the presence of ventricular chambers dilatation and contractile dysfunction leading to heart failure (HF) [1]. Thus, rather than a single nosographic entity, DCM can be conceived as a morpho-functional phenotype representing the end stage of common pathways possibly deriving from a range of diverse underlying causes (infectious, metabolic, inflammatory, toxic, genetic) [2]. If an etiology is not deemed despite a careful clinical evaluation and instrumental diagnostic work-up, DCM is considered as idiopathic DCM (iDCM).

Among HF cases, subjects presenting with iDCM are at least 10 years younger than other etiologic cohorts (ischemic, hypertensive, and valvular) and more critically ill (75% in NYHA class III-IV). They carry a mortality rate comparable to valvular disease (≈65% at 5 years) that is only inferior to ischemic cardiomyopathy with reduced ejection fraction [3]. In this context, non-pharmacological approaches such as electrical device implants [4], mechanical circulatory supports [5,6], surgical corrections [7], and stem cell based therapies [8] have found space showing different degrees of feasibility and efficacy on long-term survival and life-quality improvement. Nevertheless, the current pharmacological mainstay for iDCM does not significantly differ from the general HF population in light of the frequent inclusion of such a subgroup in large clinical trials [9].

The recognition of this paradox may require consideration of iDCM as an independent single entity, as well as the revaluation of the role of drugs that are not employed as first-line therapy in iDCM. This backward approach allows us to resurrect interest in neglected drugs and, through an inductive reasoning, to reach a deeper understanding of iDCM abnormalities and pathophysiological mechanisms.

## 2. Methods

We searched the Pubmed Library (last access 1 June 2017) for articles published between 1985 and 2016 in English with full text available and reference lists of related papers. Terms employed in the query were “idiopathic dilated cardiomyopathy” and “clinical trial”, either alone or together in different combinations. Only post-hoc analysis and placebo-controlled studies were included, as well as those where the term dilated was mentioned within the text as the phenotype of either a percentage or the total population study. Exclusion criteria were: pediatric patients, device and/or stem cell based therapies, pheresis and intracoronary infusions, and acute drug effect evaluation without follow-up. In addition, in the case of the mentioned primary cause of myocardial dysfunction and organ dilatation (viral myocarditis, alcohol or drug abuse, etc.) studies were not included, as these cases can be successfully treated by exposure cessation or targeted therapies.

In light of the diverse classes of drugs, clinical heterogeneity and different methods of data reporting, a meta-analysis was unfeasible. A meta-analysis was unfeasible for several reasons: different drug classes, diverse therapeutic mainstays (pre-β blocker and β blocker eras), clinical heterogeneity (from suboptimal to severely impaired left ventricle ejection fraction), different methods of data reporting, and the unavailability of single patient data. Nevertheless, statistical significance for changes upon treatment for molecular results, morphofunctional parameters, and clinical endpoints were reported for every study. We reported descriptive statistics with charts and graphs with Microsoft Office Excel for MacOS X.

## 3. Results

### 3.1. General Study Characteristics

The query identified 33 studies published within our selected timeframe.

**Figure 1:**
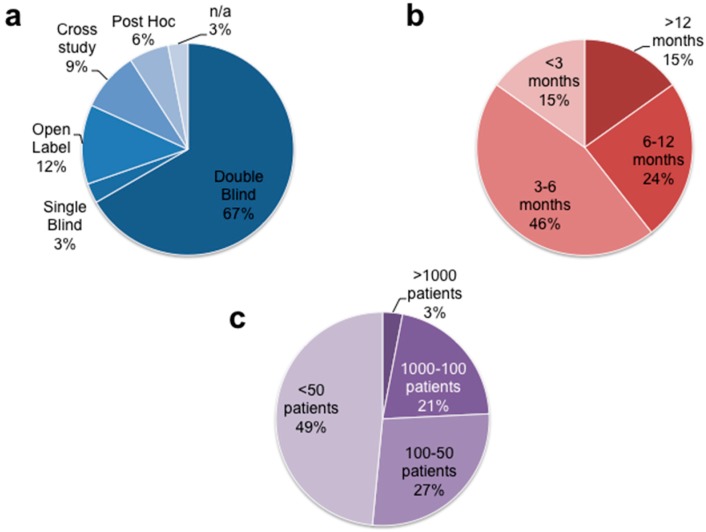
Pie charts for a comprehensive and general overview concerning features of 33 included studies: (**a**) study type; (**b**) duration; (**c**) size.

Figure 1 illustrates the distribution of size, design and follow-up length of the analyzed clinical studies [10,11,12,13,14,15,16,17,18,19,20,21,22,23,24,25,26,27,28,29,30,31,32,33,34,35,36,37,38,39,40,41,42].

Three studies were not included as we considered only the completed, the largest, and the most comprehensive dataset of a study to our knowledge in spite of respective ongoing reports.

**Figure 2:**
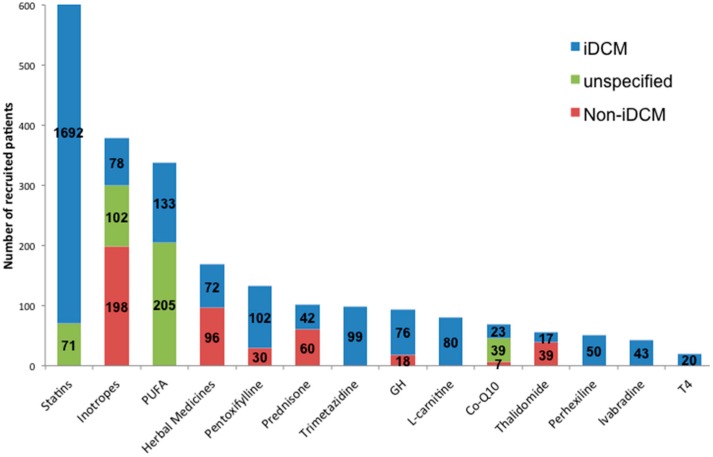
Bar graph showing the total number of recruited patients per drug class. Of note, the unspecified and non-iDCM groups represent heterogeneous population composed of ischemic, valvular, hypertensive, peripartum, post viral myocarditis cardiomyopathy, and an undisclosed number of iDCM subjects.

As shown in Figure 2, among 3392 patients and at least 2527 subjects (74.5%) were classified as iDCM or non-ischemic DCM. Only 8 studies (24%) recruited more than 100 patients, and 5 (15%) had a mean follow-up duration longer than 12 months. These characteristics could have negatively affected the accomplishment of a robust statistical significance and a hard end-point. When reported, the highest LVEF threshold was 47% for patient recruitment; nevertheless, the average LVEF was variable among the studies (ranging from 18% to 40%). Possible ischemic etiology was assessed either by scintigraphic, angiographic, or electrocardiographic methods, functional tests (6 min walking test), a clinical presentation, or anamnestic evaluation. Table 1 shows the individual study characteristics.

### 3.2. Overall Findings

**Figure 3:**
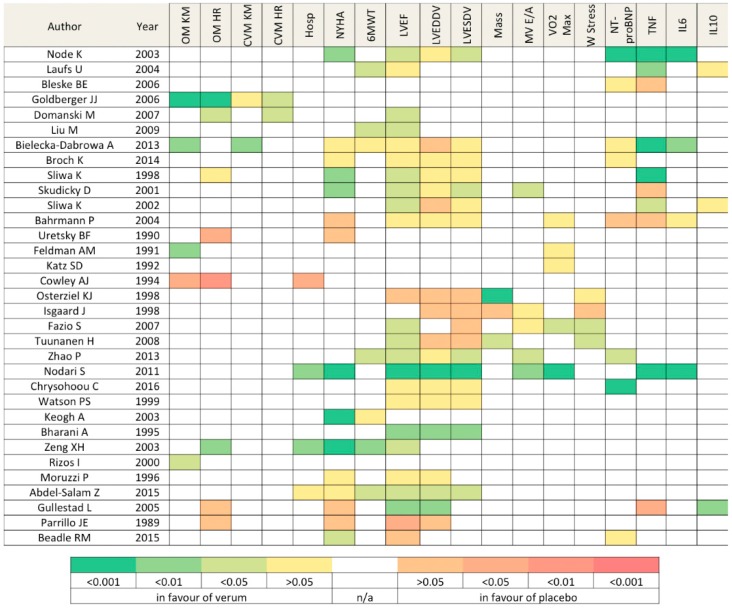
Heatmap reporting statistical significance of molecular results, morphofunctional parameters, and clinical endopoints in 33 clinical studies. Abbreviations—(OM KM) overall mortality Kaplan-Meier, (OM HR) overall mortality Hazard Ratio, (CVM KM) cardiovascular mortality Kaplan-Meier, (CVM HR) cardiovascular mortality Hazard Ratio, (Hosp) hospitalization, (NYHA) NYHA functional class, (6MWT) 6-min walking test, (LVEF) left ventricle ejection fraction, (LVEDDV) left ventricle end-diastolic dimension/volume, (LVESDV) left ventricle end-systolic dimension/volume, (Mass) Cardiac Mass, (MV E/A) Mitral valve E/A ratio, (VO2 Max), maximum oxygen uptake, (W Stress) wall stress.

Heatmap in Figure 3 offers a general overview of the overall findings among selected studies.

The drug effect on biomarkers revealed a general positive trend of inflammation resolution for several classes (statins, pentoxifylline, PUFAs): TNFα plasma concentration showed up to a threefold decrease with treatment [18]. In contrast, thalidomide increased its concentration. NT-proBNP levels decreased in the aforementioned drug classes too [10,12,16,17,30,32].

Echocardiographic assessment revealed a substantial or even significant benefit for LVEF in the verum cohorts with the exception of perhexelline and corticosteroids. However, data were uncomparable among groups in light of the various available pharmacologic treatments at different timepoints (pre-β-blocker and β-blocker era). Notably, LVEF improvement was more frequently associated with LV end-systolic dimension reductions rather than the end-diastolic, suggesting a more distinct role on contractility instead of sole remodeling. Functional improvement (either assessed by a 6 min-walking test or an NYHA class) was detected in the majority of the studies, particularly in medications related to energetics improvement (CoQ10).

Thirteen studies (39%) reported results on hard clinical end-points, such as hospitalization rates and cardiovascular or overall mortalities. This low response rate reflects a series of factors: a short mean follow-up, low prescription prevalence, drug costs, and the heterogenous clinical status of patients recruited in different trials. Patients treated with statins had significantly higher survival rates (≈75% vs. 40% at 5-year follow-up *p* < 0.01) and the largest study analyzed proved a significant protection despite a lack of ischemic etiology (Adjusted CV Mortality HR = 0.42, 95% 0.18–0.95 *p* < 0.04). Steroids and thalidomide showed a reduced, yet non-significant, survival rate. Not surprisingly, long PDEi treatments were associated with higher mortality rates. Of note, the benefit (survival rate) occurring at the earliest timepoint (2 months) was found for treatment with the natural plant extract Berberine (91% vs. 86% *p* < 0.01).

## 4. Discussion

Positive and negative results collected from different clinical trials allowed us to reevaluate current treatments and highlight specific molecular pathways in the management of HF. This specular approach, in opposition to the traditional drug development pipeline, provides evidence-based insights and immediate interpretation.

As postulated by Chien nearly 20 years ago, dilated cardiomyopathy relies on four biological components which are intimately linked to each other (biomechanical stimuli, cytoskeletal signaling, myocyte survival, and calcium cycling) [43]. Since a remarkable body of literature on iDCM has grown in recent years, we can revisit such a biological equation.

### 4.1. Not All Statins Were Created Equal

Being commonly prescribed, studies on statins provide the most robust dataset. Statins differ among each other for several pharmacokinetics criteria, and lipophilicity is a critical determinant. Lipophilic statins (Atorvastin, Simvastatin, i.e.,) can passively diffuse the membrane of extrahepatic cells. This ability supports the findings by Tsutamoto who demonstrated a superior clinical improvement in DCM patients with Atorvastatin compared to a hydrophilic one (Rosuvastatin) [44]. Statins, along with their well-known lipid-lowering effect, have pleiotropic effects due to the inhibition of 3-hydroxy-3methyl-glutaryl-coenzyme A (HMG-CoA) reductase. Such blockade decreases mevalonate synthesis and, ultimately, farnesylpyrophosphate biosynthesis, a critical biochemical crossroad shared by G-protein Rho and its numerous related subfamily members (Rac, ROCK, Ras), as described by Oesterle [45]. Rho downstream effectors lead to cell proliferation, differentiation, and cytoskeletal changes directly affecting cell geometry, integrity, adhesion, and stability [46,47,48]. In addition, statins can relieve inflammatory signaling by modulating Rac1 and peroxisome proliferator activated receptors (PPARs), which in turn decrease ROS and NADPH oxidase activity and TNF-α concentrations respectively [49,50].

### 4.2. Inflammation Resolution: Is It Worth?

As anticipated, systemic, and local inflammation have been viewed as pivotal components of myocardial dysfunction. In the past a plethora of putative inflammatory mediators has been evaluated. Among them, TNFα is recognized as a major effector of myocardial damage and considered as a reliable HF biomarker for a number of reasons [51,52,53,54,55,56]. In addition to the reported increase in tissue and plasma TNFα concentration in patients with HF and iDCM, cardiac-specific transgenic mice overexpressing TNFα recapitulated the human pathophysiology of DCM with four-chamber dilatation, myocyte hypertrophy, increased fibrosis, diminished β-adrenergic responsiveness, and premature death [57]. These findings composed a solid biological rationale to target TNFα and were sufficient and appropriate to justify the clinical sperimentation of a monoclonal antibody or a decoy receptor (infliximab and etanercept). Nevertheless, the complete biological abrogation of this cytokine did not lead to any significant clinical benefit, suggesting additional underestimated compensatory effects by TNFα and a more complex biological role in HF pathophysiology [58,59,60].

Antinflammatory therapies such as thalidomide have had minimal clinical application in iDCM management and steroideal drugs are advisable only to resolve viral driven damage in the setting of positive myocardial lymphocytic infilitration or HLA hyperexpression [61]. In contrast, a xantine derivative (namely pentoxifylline) was employed in four different trials for a total of 151 randomized iDCM subjects for antagonizing TNFα. Results were promising for reducing apoptotic stimuli and inflammatory biomarkers [20], translating into a clinical improvement, but were still insufficient to grant its use in the general DCM population, although worthy for starting a larger clinical trial. These outcomes cannot be simply linked to the inflammatory resolution; in fact, pentoxifyilline has a positive inotropic and vasodilatatory activity above the other effects. This aspect is not marginal, especially in the DCM setting.

### 4.3. Inotropism Manipulation: A Double-Edged Knife

Inotropic agents are engulfed together in light of their positive effect on myocardial contractility. Their various mechanisms of action are extensively described elsewhere [62,63]. In the race for HF management, such agents have gained momentum in the past, despite not meeting the expectations they generated during their introduction. Among them, the “calcium mobilizers” class is a heterogenous group of drugs that modulate calcium dynamics through different mechanisms with degrees of specificity for molecular targets ultimately leading to contractile improvements [64]. Over the last three decades, several of these drugs have been developed, but only few have found a clinical application, especially in the setting of acute HF. In fact, an intracellular calcium overload is detrimental for functional and survival reasons exceeding the advantages on the contractile apparatus. This double-edged knife effect applies to the decompensated heart too where calcium overload cannot be handled properly by the calcium handling proteins [65,66,67]. In the setting of ischemic cardiomyopathy, an insufficient blood supply does not allow a proper ATP availability to sustain ATP-dependent calcium handling pumps, justifying the use of this inotropic mean in the unlikely case of first-line therapies’ unresponsiveness (diuretics). Interestingly, Dec reported the significant difference between the idiopathic dilated phenotype and ischemic etiology in patients receiving oral enoximone: only 5% of ischemic patients were alive at 18 months follow-up versus 66% of DCM [68]. This is a dramatic divergence between the DCM subgroup and the general HF population in terms of clinical long-term endpoint. It thus appears as a valid reason to reconsider cardiac inotropes for medium-termed treatments and for this specific subgroup, in spite of their general contemporary minimal employment.

### 4.4. Mitochondrion: A Hijacked Powerhouse

In this scenario, cardiac energetics must be also considered, as both contraction and relaxation heavily rely on ATP availability. It is now broadly accepted that mitochondria are involved in a wide range of diseases, including HF [69]. Of note, among nearly 40 target genes associated with DCM [70], TAZ (tafazzin) is enlisted too and it encodes for a component of the mitochondrial inner membrane [71]. The myocardial metabolism of iDCM is severely impaired, with energetic starvation, and is characterized both at transcriptional and functional levels by free fatty acid (FFA) metabolism reduction and glucose metabolism induction, whose use is energetically more efficient [72,73]. Interestingly, insulin resistance is a relatively common comorbidity in iDCM patients and it might suggest an interference in glucose uptake too [74]. Whether HF precedes metabolic alterations or vice versa remains a “chicken or egg” dilemma. Starting from this paradox, a series of clinical trials was started and here reported. Coenzyme Q10 and l-carnitine represent two valid dietary supplementations that could have a therapeutic role by enhancing mitochondrial activity either by favoring the electron transport chain between complex II and III or increasing FFA as a substrate, respectively. The former has been extensively described in a recent review [75], and in our review only the NYHA functional class appeared to significantly improve. Instead, l-carnitine was found to significantly decrease the overall mortality in iDCM patients (3% vs. 18% at 3 year follow-up). Ironically, trimetazidine, whose mechanism of action is FFA oxidation blockade, was found to be beneficial on cardiac function and clinical symptoms in shorter observation times too. Perhexelline, which is conceptually opposite to l-carnitine, improved the NYHA class too. All in all, such findings should be confirmed and implemented with future, larger, and longer studies. Nevertheless, reported trivial findings from conceptually different rationales still question whether these metabolic alterations are putative pathologic mechanisms to be tackled or compensatory ones to be enhanced.

### 4.5. Growth Hormone: New Bricks for a Crumbling Heart?

Finally, a component of DCM progression included in the above mentioned equation is myocyte apoptosis. It is widely accepted that cell loss is a critical determinant of HF [76,77]. Over time, a net depletion of the myocyte count contributes to the cardiac dysfunction generally observed with aging or in certain cardiomyopathies. As reported by Pluess, iDCM hearts show high variability in cell size, as well as prominent fibrous deposition affecting the 3D structure leading to abnormal cell-to-cell contact. Such architectural changes constitute a proarrhythmogenic anatomical substrate and bring the cell unit to a sub-optimal mechanical coordination with neighbouring cells [78]. Independently from the magnitude of these events, growth hormone (GH) represents a valid and applealing option to counteract such defects by suppressing apoptosis and favoring cell proliferation [79]. In 1996, a non-placebo-controlled clinical study demonstrated remarkable improvements in systolic and diastolic function (LVEF increase, LV end diastolic and systolic dimension reduction, E/A ratio improvement) over a 3 month treatment [80]. These results were not met by subsequent clinical placebo-controlled studies, leaving many unanswered questions concerning its use as drug cost-effectiveness, the administration method (continuous vs. pulsitile), treatment duration (>6 months), and possible colonic neoplastic evolution [81]. If GH is recommendable in case of deficiency [82], its use in iDCM remains a matter or investigation and evaluation in light, also, of a small clinical study with positive results testing the cardiovascular effect of octreotide [83], a somatostatin analog [84].

### 4.6. A Challenging Equation

**Figure 4:**
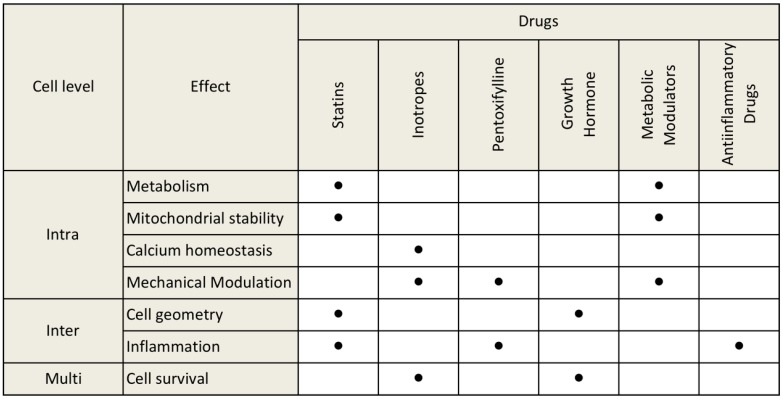
Summary of pleiotropic and overlapping effects of drug classes towards putative pathologic driving mechanisms of iDCM.

Cell stability, inflammatory status, metabolic depletion, mechanical modulation, calcium dynamics, and cell survival compose a tangled interplay of factors intimately linked with each other in the determinism of iDCM development and progression. Moreover, as anticipated, several drugs reported in this study have pleiotropic and overlapping effects over a series of molecular and cell targets. In order to update this equation, Figure 4 was conceived to better summarize the results that emerged from the present work and to help future researchers and physicians to solve this jigsaw.

## 5. Conclusions

In conclusion, the present study highlights putative therapeutic targets that can be employed for the treatment of iDCM based on previous clinical trials. This goal can be reached through several efforts. As adviced by Bierer, data authorship policies can be a future incentive for data sharing by promoting the generation of a larger database merging past clinical studies’ results [85]. In addition, future studies are encouraged to be multricentric and multimodal in light of the specific phenotype of iDCM and the urge to assess drug effectiveness at cell, tissue, and whole organ levels. Furthermore, in the era of precision medicine, the use of new algorithms based on novel prognostic biomarkers will help clinicians to tailor the most suitable therapy for such a heterogenous population. Lastly, the identification of molecular targets can lead to drug repurposing and the development of new ones in the near future. By adopting these solutions, medicine will replenish its pharmacological armamentarium in the war against heart failure [86].

## 6. Limitations

Limitations of the present study are as follows. The search criteria are stringent in order to be specific for the idiopathic DCM phenotype, and therefore a number of studies were not included because of heterogenous nomenclature. The use of non-ischemic cardiomyopathy wording allows for the exclusion of coronaropathies, but does not distinguish between other etiologies (hypertensive, valvular, inflammatory, toxic, and idiopathic). The pediatric population was not included in the current study as this peculiar subgroup is generally separated from the adult population of clinical trials. The report of statistical significance does not provide any information regarding the magnitude of benefit or the damage derived from treatment.

## Figures and Tables

**Table 1:** Clinical studies targeted for dilated cardiomyopathy pharmacological management.

Author	Year	No. of Patients iDCM/Total (%)	Study Type and Design	Active Drug Target	Follow-up (Months)	LVEF %	
						Threshold	Baseline Average
**Statins**							
Node K [10]	2003	48/48 (100%)	Double blind	Simvastatin (10 mg/day)	3	40	34
Laufs U [11]	2004	15/15 (100%)	Double blind	Cerivastatin (0.4 mg/day)	5	n/a	40
Bleske BE [12]	2006	15/15 (100%)	Crossed	Atorvastatin (80 mg/day)	3	45	25
Goldberger JJ [13]	2006	458/458 (100%)	Post hoc analysis	Any statin at any dosage	24	35	20
Domanski M [14]	2007	1024/1024 (100%)	Post hoc analysis	Any statin at any dosage	24	35	25
Liu M [15]	2009	64/64 (100%)	Double blind	Atorvastatin (10mg/day)	3	40	35
Bielecka-Dabrowa A [16]	2013	68/68 (100%)	Open	Atorvastatin (10 or 20 mg/day)	60	n/a	32
Broch K [17]	2014	71 unspecified	Double blind	Rosuvastatin (10 mg/day)	6	40	36
**Pentoxifylline**							
Sliwa K [18]	1998	28/28 (100%)	Double blind	Pentoxifylline (400 mg/tid)	6	40	22
Skudicky D [19]	2001	39/39 (100%)	Double blind	Pentoxifylline (400 mg/tid)	6	40	24
Sliwa K [20]	2002	18/18 (100%)	Double blind	Pentoxifylline (400 mg/tid)	1	40	16
Bahrmann P [21]	2004	17/47 (36.2%)	Double blind	Pentoxifylline (600 mg/bid)	6	40	29
**Inotropes**							
Uretsky BF [22]	1990	102 unspecified	Double blind	Enoximone (100 or 150 mg/tid)	4	n/a	22
Feldman AM [23]	1991	38/76 (50%)	Double blind	Vesnarinone (60 mg/day)	3	n/a	24
Katz SD [24]	1992	14/49 (28.6%)	Double blind	Pimobendan (5 or 10 mg/day)	3	n/a	19
Cowley AJ [25]	1994	26/151 (16.6%)	Double blind	Enoximone (50 or 100 mg/tid)	12	n/a	n/a
**Growth Hormone (GH)**							
Osterziel KJ [26]	1998	50/50 (100%)	Double blind	rhGH subq (2 IU/qd)	3	45	26
Isgaard J [27]	1998	13/22 (59.1%)	Double blind	rhGH subq (to 4 IU/qd)	3	45	30
Fazio S [28]	2007	13/22 (59.1%)	Double blind	rhGH subq (to 4 IU/qod)	3	40	32
**Trimetazidine**							
Tuunanen H [29]	2008	19/19 (100%)	Single blind	Trimetazidine (35 mg/bid)	3	47	31
Zhao P [30]	2013	80/80 (100%)	Double blind	Trimetazidine(20mg/tid)	6	40	34
**Polyunsaturated Fatty Acids (PUFAs)**							
Nodari S [31]	2011	133/133 (100%)	Double blind	EPA/DHA850 mg/bid	12	45	36
Chrysohoou C [32]	2016	205 unspecified	Open	PUFA 1000 mg/day	6	40	28
**CoQ10**							
Watson PS [33]	1999	23/30 (76.7%)	Cross	CoQ10 (33 mg/tid)	3	35	26
Keogh A [34]	2003	39 unspecified	Double blind	CoQ10 (150 mg/day)	3	40	n/a
**Herbal Medications**							
Bharani A [35]	1995	10/12 (83.3%)	Cross	*Terminalia Arjuna* (500 mg/tid)	0.5	n/a	30
Zeng XH [36]	2003	62/156 (39.8%)	Double blind	Berberine (up to 0.5 g/qid)	2	n/a	22
**L-carnitine**							
Rizos I [37]	2000	80/80 (100%)	Open	L-carnitine (2 g/day)	34	n/a	27
**Levotyroxine (T4)**							
Moruzzi P [38]	1996	20/20 (100%)	n/a	Levotyroxine (100 ug/day)	3	40	30
**Ivabradine**							
Abdel-Salam Z [39]	2015	43/43 (100%)	Double blind	Ivabradine(2.5 mg/tid)	3	40	34
**Thalidomide**							
Gullestad L [40]	2005	17/56 (30.4%)	Double blind	Thalidomide (200 mg/qd)	3	40	24
**Steroids**							
Parrillo JE [41]	1989	42/102 (41.2%)	Open	Prednisone (60 mg/day) for 3 months	15	35	18
**Perhexelline**							
Beadle RM [42]	2015	50/50 (100%)	Double blind	Perhexelline (200 mg/day)	2	40	27
